# Parathyroid hormone reference ranges in healthy individuals classified by vitamin D status

**DOI:** 10.1007/s40618-019-01075-w

**Published:** 2019-07-04

**Authors:** N. Yalla, G. Bobba, G. Guo, A. Stankiewicz, R. Ostlund

**Affiliations:** 1 Washington University, St. Louis, MO USA; 2 Roche Diagnostics International Ltd, Rotkreuz, Switzerland; 3 Roche Diagnostics Inc., Indianapolis, IN USA

**Keywords:** Parathyroid hormone, Vitamin D, 25-Hydroxyvitamin D (25(OH)D), Reference range

## Abstract

**Purpose:**

Parathyroid hormone (PTH) concentrations are routinely measured in the diagnosis and management of bone and kidney diseases, but reference ranges can be overestimated if determined in otherwise healthy individuals for whom vitamin D deficiency was not evaluated. We establish PTH reference ranges in apparently healthy, normocalcemic, normophosphatemic individuals categorized by 25-hydroxyvitamin D (25(OH)D) status using the Elecsys^®^ PTH (**cobas e** 601) and Elecsys^®^ Vitamin D total II electrochemiluminescence immunoassays (**cobas e** 411).

**Methods:**

This prospective, non-interventional study measured PTH in serum from 653 apparently healthy adults [56.7% female; 68.2% white/Caucasian; 28.6% African American; median age 44 years (range 21–83)] from three diverse geographic sites across the USA during summer and winter months. Subjects were classified by concomitant vitamin D sufficiency (≥ 30 ng/mL), insufficiency (> 20 to < 30 ng/mL) or deficiency (≤ 20 ng/mL).

**Results:**

In vitamin D sufficiency, median PTH was 31.9 pg/mL [range (2.5th–97.5th percentile) 17.9–58.6] compared with 35.5 pg/mL (17.0–60.4) for insufficiency, and 39.8 pg/mL (19.5–86.4) for deficiency. A significant inverse relationship was found between PTH and 25(OH)D (*P *< 0.001). After accounting for vitamin D, potential effects of race or season as covariates were relatively small or absent.

**Conclusions:**

Upper reference limits (URL) for PTH in vitamin D sufficiency/insufficiency were similar and lower than current values. Clinically important PTH elevations were observed in vitamin D deficiency, where revised reference ranges with a higher URL may be appropriate. These data may help to distinguish vitamin D-related PTH elevations from other causes [e.g., primary (normocalcemic) or secondary hyperparathyroidism].

## Introduction

Parathyroid hormone (PTH) and vitamin D are major regulators of mineral metabolism and form a tightly controlled feedback cycle; PTH stimulates 1,25-dihydroxyvitamin D synthesis, which in turn exerts a negative feedback on the parathyroid glands [1, 2]. PTH concentrations are routinely measured in the diagnosis and management of bone and mineral disease and chronic kidney disease (CKD) [3, 4]; however, the target range for PTH may vary significantly depending on vitamin D status, defined as the sum of 25(OH)D_2_ and 25(OH)D_3_ circulating levels [5, 6]. Vitamin D deficiency occurs frequently in the general population, with an estimated prevalence of 41.6% among US adults, increasing to 82.1% in black individuals, and elevated PTH secretion is associated with vitamin D deficiency [1, 2, 7]. Given the interdependency of PTH and vitamin D, reference ranges for PTH may be overestimated if determined in a population of otherwise healthy individuals for whom vitamin D deficiency was not evaluated. Accurate reference ranges for PTH may facilitate more reliable diagnosis and management of conditions such as primary hyperparathyroidism and end-stage renal disease [8–11].

We aimed to determine accurate reference ranges for PTH by vitamin D status in apparently healthy individuals. Serum 25(OH)D (sum of D_2_ and D_3_) and intact PTH were measured using the Elecsys^®^ Vitamin D total II and Elecsys^®^ PTH electrochemiluminescence assays, respectively (Roche Diagnostics GmbH, Mannheim, Germany) [12, 13].

## Methods

### Study design and participants

We conducted a multicenter, prospective, non-interventional study to determine reference ranges for intact PTH in apparently healthy, normocalcemic, normophosphatemic individuals according to vitamin D status, using the Elecsys^®^ PTH and Elecsys^®^ Vitamin D total II electrochemiluminescence immunoassays. Volunteers were enrolled at three geographic sites across the USA (Century Clinical Research Inc., Daytona Beach, Florida, USA; NB Research, Indianapolis, Indiana, USA; Prism Research, LLC., St Paul, Minnesota, USA). Prior to study initiation, ethical approval was obtained from relevant institutional review boards. The study was conducted in accordance with the principles of the Declaration of Helsinki and International Conference on Harmonisation guidelines for Good Clinical Practice; all participants provided written informed consent.

Apparently, healthy individuals aged ≥ 21 years with body mass index (BMI) 18–30 kg/m^2^ were enrolled. Key inclusion criteria were: calcium (≤ 60 years, 8.6–10.0 mg/dL; > 60 years, 8.8–10.2 mg/dL), phosphate (2.5–4.5 mg/dL), and creatinine (female 0.51–0.95 mg/dL; male 0.67–1.17 mg/dL), based on medical history and confirmatory testing; geographic location within ± 2° latitude or 138 miles north/south of collection site for ≥ 4 weeks. Key exclusion criteria were pregnancy (self-declared/≤ 12 months); breastfeeding or lactation (≤ 3 months); endocrine/metabolic disease known to affect vitamin D metabolism or interfering with bone metabolism; abnormal calcium, including hypocalcemia, hypocalciuria, hypophosphatemia, or hypercalcemia caused by primary hyperparathyroidism, vitamin D overdose/intoxication, or cancer; bariatric surgery; vigorous exercise (> 2 h/day); hospitalization/immobilization > 7 days (≤ 3 months); bone fracture (≤ 3 months). Individuals taking the following supplements or drugs influencing bone and calcium/phosphorus metabolism were excluded: PTH analogs and/or modified PTH compound; other anabolic medication; anticonvulsants; antiresorptive drugs, e.g., bisphosphonates, calcitonin; hormone replacement therapy; calcium carbonate; diuretics; fluoride; glucocorticoids; high-dose vitamin D supplements (> 1000 IU/day).

### Sample collection and storage

Blood samples were collected during summer (August 2015 and June 2016) and winter months (December 2015 and February 2016); an individual could only contribute an evaluable sample during one collection period. Aliquots of serum samples were prepared within 2 h of venipuncture and immediately frozen for storage at − 70 °C or colder. De-identified samples were tested at a central laboratory (Washington University, USA).

### Determination of PTH reference ranges

Serum PTH and 25(OH)D concentrations were measured with the Elecsys^®^ PTH and Elecsys^®^ Vitamin D total II electrochemiluminescence immunoassays on the **cobas e** 601 and **cobas e** 411 analyzers, respectively. The Elecsys^®^ PTH assay employs the sandwich test principle, whereas the Elecsys^®^ Vitamin D total II assay employs a competition principle. Both assays have turnaround times of less than 30 min. The Elecsys^®^ Vitamin D total II assay has been standardized using internal standards, which are traceable to the ID-LC–MS/MS 25-hydroxyvitamin D Reference Measurement Procedure [14, 15]. The ID-LC–MS/MS is traceable to the National Institute of Standards and Technology Standard Reference Materials 2972 [16]. The Elecsys^®^ PTH assay was standardized against a commercial PTH test (RIA) [13]. Recovery of the NIBSC 95/646 (WHO) standard was assessed using serial dilutions in human serum covering the measuring range (40–4000 pg/mL) on multiple analyzers, and resulted in a mean recovery of 100% ± 4%. Calcium, phosphate and creatinine concentrations were measured with the Calcium Gen 2, Phosphate Inorganic version 2 and Creatinine Plus version 2 assays (respectively; Roche Diagnostics GmbH, Mannheim, Germany), on the **cobas c** 501 analyzer. Assays were performed according to manufacturer instructions with daily QC runs.

Target sample size was 190 evaluable individuals per site (minimum *n *= 146). PTH reference ranges [medians 2.5th–97.5th percentiles and 95% confidence intervals (CI) of the upper (URL) and lower reference limits (LRL)] were calculated using standard nonparametric analyses, according to CLSI EP28-A3c guidelines [17]. PTH reference ranges were determined for three cohorts by vitamin D status: deficiency (25(OH)D ≤ 20 ng/mL), insufficiency (25(OH)D > 20 to < 30 ng/mL), and sufficiency (25(OH)D ≥ 30 ng/mL), per national guidelines [18, 19].

### Statistical analyses

Bivariate associations were evaluated using both Pearson’s correlation (*R*) and Kendall’s Tau. Kruskal–Wallis analysis was used as a non-parametric approach to assess the statistical significance of differences in serum PTH measurements by 25(OH)D categories and by demographic variables, including race and site. When overall differences were found, the significance of pairwise comparisons was determined using the Dwass–Steel–Critchlow–Fligner (DSCF) multiple comparison procedure. General linear regression modeling was used to assess the association between PTH and 25(OH)D measurements in serum. Natural log transformations were performed on serum PTH measurements to normalize the distribution and reduce the influence of outliers when calculating Pearson’s *R* and analyzing general linear regression models. Covariates of race, site, or season were added to the general linear regression model of PTH = 25(OH)D to assess their impact on PTH in serum.

## Results

### Analysis population

**Fig. 1:**
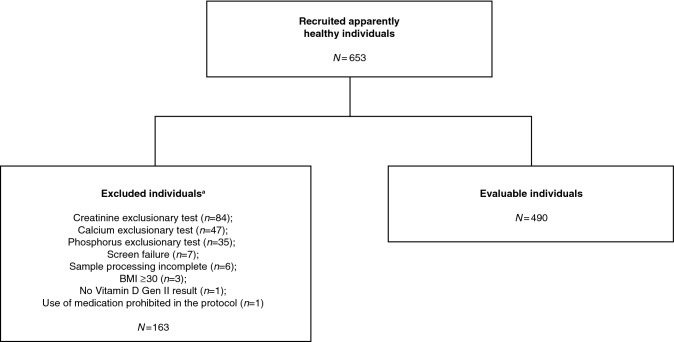
Participant flow diagram. ^a^Some individuals met multiple exclusion criteria (i.e., some participants had levels of both calcium and phosphorus that exceeded requirements). *BMI* body mass index

Samples were collected from 653 individuals; 490 evaluable serum samples were included in the analyses (Fig. 1). A total of 163 individuals were excluded prior to analyses; the most frequent reason for exclusion was creatinine level outside the accepted range during exclusionary testing (*n *= 84). In the evaluable cohort, median (range) age was 44 (21–83) years and 278 (56.7%) individuals were female; 334 (68.2%) were white/Caucasian, 140 (28.6%) were black/African American, and 5 (1.0%) were Asian.

**Fig. 2:**
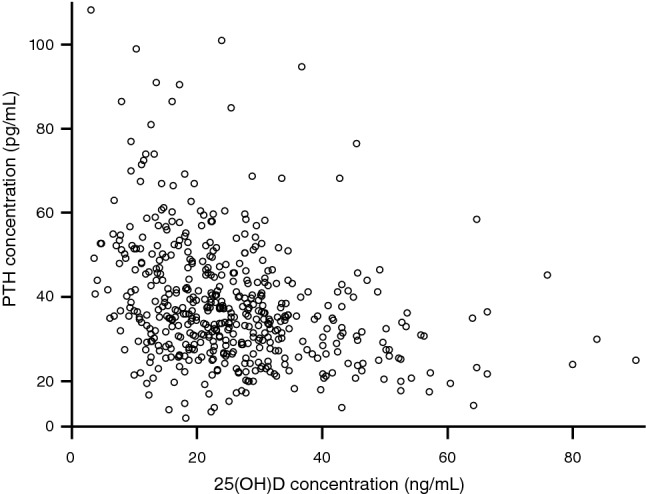
Inverse relationship between PTH and 25(OH)D in a USA reference population. *25(OH)D* 25-hydroxyvitamin D, *PTH* parathyroid hormone

Across all samples, median (2.5th–97.5th percentile) serum PTH concentration was 35.5 pg/mL [17.3 (95% CI 13.8–19.5) to 76.4 (95% CI 68.8–90.9) pg/mL]. Median PTH concentrations and associated URLs decreased with increasing 25(OH)D concentration (Table 1); among vitamin D-deficient individuals PTH levels ranged from 19.5 to 86.4 pg/mL, whereas among vitamin D-replete individuals PTH levels ranged from 17.9 to 58.6 pg/mL. Analysis with Kendall’s Tau showed a significant inverse relationship between PTH and 25(OH)D (Kendall coefficient − 0.225, *P *< 0.001; Pearson’s *R* = − 0.302, *P *< 0.0001; Fig. 2). Kruskal–Wallis analysis revealed a statistically significant difference in serum PTH level between the three vitamin D clinical categories (25(OH)D ≤ 20, > 20 to < 30, and ≥ 30 ng/mL). A further analysis of multiple comparisons based on pairwise rankings revealed significant differences between each pair (25(OH)D ≥ 30 vs > 20 to < 30 ng/mL, *P *= 0.008; ≥ 30 vs ≤ 20 ng/mL, *P *< 0.0001; and > 20 to < 30 vs ≤ 20 ng/mL, *P *= 0.001).

**Table 1:** PTH reference ranges according to season of collection and vitamin D category (25(OH)D ≤ 20, > 20 to < 30, and ≥ 30 ng/mL)

Season	Median PTH concentration, pg/mL											
	25(OH)D concentration ≤ 20 ng/mL			25(OH)D concentration > 20 to < 30 ng/mL^a^			25(OH)D concentration ≥ 30 ng/mL			Total		
	Median (*n*)	Percentile (95% CI)		Median (*n*)	Percentile (95% CI)		Median (*n*)	Percentile (95% CI)		Median (*n*)	Percentile (95% CI)	
		2.5th	97.5th		2.5th	97.5th		2.5th	97.5th		2.5th	97.5th
Summer	39.2 (71)	16.7 (NC)	74.1 (NC)	37.4 (81)	20.1 (NC)	60.4 (NC)	31.6 (94)	14.3 (NC)	51.9 (NC)	35.1 (246)	18.1 (13.2–20.4)	74.0 (60.7–101.0)
Winter	40.0 (112)	19.5 (NC)	86.6 (NC)	33.0 (85)	13.8 (NC)	60.4 (NC)	33.7 (47)	17.7 (NC)	68.3 (NC)	35.8 (244)	17.2 (11.3–19.4)	77.0 (68.8–99.0)
Total	39.8 (183)	19.5 (11.3–22.2)	86.4 (74.0–108.3)	35.5 (166)	17.0 (12.8–20.1)	60.4 (59.5–325.7)	31.9 (141)	17.9 (13.7–20.6)	58.6 (51.0–94.9)	35.5 (490)	17.3 (13.8–19.5)	76.4 (68.8–90.9)

*25(OH)D* 25-hydroxyvitamin D, *CI* confidence interval, *NC* not calculated, *PTH* parathyroid hormone

^a^One individual within the > 20 to < 30 ng/mL vitamin D category had a median PTH level of 325.66 pg/mL; for transparency, we have included the outlier in calculating the median value

**Fig. 3:**
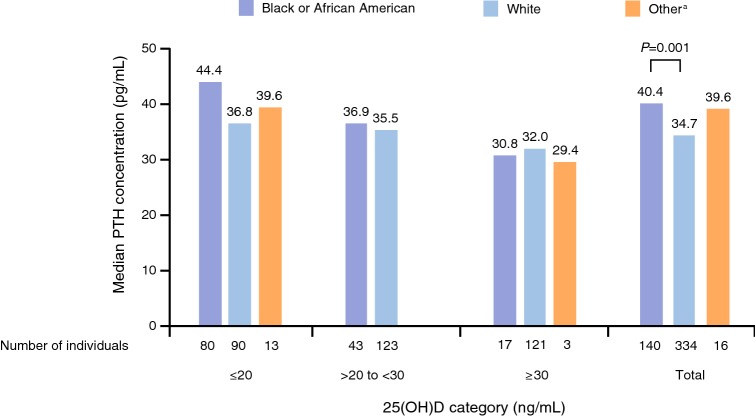
PTH levels by vitamin D category (25(OH)D ≤ 20, > 20 to < 30, and ≥ 30 ng/mL) for each race. ^a^Sample cohorts for Asian, other, and more than one race were combined into one category [presented here as ‘other’ (*n *= 16)] due to small sample sizes for each 25(OH)D category. *25(OH)D* 25-hydroxyvitamin D, *PTH* parathyroid hormone

Median PTH concentration was significantly higher in black/African American individuals (40.4 pg/mL) than in white individuals (34.7 pg/mL; *P *= 0.001; Fig. 3), with the greatest between-race difference observed among individuals in the ≤ 20 ng/mL 25(OH)D category. Fewer black/African American individuals were in the > 20 to < 30 and ≥ 30 ng/mL vitamin D categories compared with white individuals (25.9% vs 74.1% and 12.1% vs 85.8%, respectively; Fig. 3), and accordingly, median 25(OH)D levels were highest in white individuals (26.7 ng/mL) compared with black/African American (17.2 ng/mL; *P *= 0.0001) and other individuals (15.9 ng/mL; *P *= 0.0017).

### Categorical analysis

Kruskal–Wallis analyses showed that any additional effects of race or season as covariates were relatively small or absent. No significant differences were found between black/African American versus other or white versus other. No statistically significant relationship was found between PTH level and sample collection season.

Median serum PTH concentrations (2.5th–97.5th percentile) were significantly different between Indianapolis versus St Paul (*P *= 0.01) and Indianapolis versus Daytona Beach (*P *= 0.0007): in the overall population, values were 33.3 pg/mL (17.7–69.2 pg/mL) for Daytona Beach, 37.6 pg/mL (16.7–86.4 pg/mL) for Indianapolis, and 35.2 pg/mL (17.0–66.6 pg/mL) for St Paul. No significant difference was observed between St Paul and Daytona Beach (North vs South USA).

### Linear regression modeling

Linear modeling confirmed the highly significant relationship between PTH and vitamin D status (according to 25(OH)D; *P *< 0.0001; Table 2). Using the model log(PTH) = 25(OH)D + race + 25(OH)D × race, significant effects on serum PTH were observed for the variables 25(OH)D status (*P *= 0.005), race (*P *= 0.01), and the interaction between race and 25(OH)D status (*P *= 0.03). The variable race did not have a significant effect on serum PTH concentration when the interaction variable 25(OH)D × race was not included in the model, i.e., log(PTH) = 25(OH)D + race (*P *= 0.18). A further analysis was done to evaluate 25(OH)D status by race, and it showed that serum 25(OH)D concentrations were significantly higher in white individuals compared with black/African American (*P *< 0.0001) and other (*P *= 0.002) individuals.

**Table 2:** Regression modeling to estimate the effect of 25(OH)D, race, and sample collection site on PTH concentration

Parameter	Degrees of freedom	Sum-of-squares	Mean squares	*F* value	*P* value
Log PTH = 25(OH)D + race + 25(OH)D × race					
25(OH)D	1	0.98	0.98	7.81	0.0054
Race	2	1.08	0.54	4.30	0.0142
25(OH)D × race	2	0.86	0.43	4.43	0.0331
Log PTH = 25(OH)D + race					
25(OH)D	1	4.61	4.61	36.4	< 0.0001
Race	2	0.44	0.22	1.73	0.179
Log PTH = 25(OH)D + site + 25(OH)D × site					
25(OH)D	1	6.23	6.23	49.5	< 0.0001
Site	2	1.13	0.56	4.48	0.0118
25(OH)D × site	2	0.04	0.02	0.15	0.859
Log PTH = 25(OH)D + site					
25(OH)D	1	6.23	6.23	49.7	< 0.0001
Site	2	1.13	0.56	4.50	0.0116

*25(OH)D* 25-hydroxyvitamin D, *PTH* parathyroid hormone

To assess the effect of collection site on PTH level, the model log(PTH) = 25(OH)D + site + 25(OH)D × site was used. The variables 25(OH)D (*P *< 0.0001) and site (*P *= 0.01) both had significant effects on serum PTH level; however, the interaction between site and 25(OH)D did not (*P *= 0.86). Similar results were observed using the model log(PTH) = 25(OH)D + site (25(OH)D, *P *< 0.0001; site, *P *= 0.01).

## Discussion

We identified an inverse relationship between PTH and vitamin D levels in serum samples from apparently healthy individuals collected over two seasons across three geographically diverse locations in the USA. The URL for PTH was lower when determined in vitamin D-replete individuals compared with vitamin D-deficient and insufficient individuals. Using the Elecsys^®^ PTH immunoassay, we determined a PTH reference range of 17.9–58.6 pg/mL in vitamin D-replete individuals. Previous studies in vitamin D-replete Caucasian individuals have reported URLs of 64 pg/mL (Elecsys^®^) and 45.3 pg/mL (Elecsys^®^) [6, 20]. Differences in population demographics/characteristics (e.g., age, race, BMI) and preanalytical considerations (e.g., time of sample collection, fasting state of volunteer, EDTA plasma/serum), as well as site-specific factors, may contribute to between-study differences in URL [3, 4, 9, 21]. In vitamin D-deficient subjects, PTH values ranged from 19.5 to 86.4 pg/mL, demonstrating a 47% increase in the URL.

Upper reference limits for PTH in the > 20 to < 30 ng/mL and ≥ 30 ng/mL vitamin D categories were similar (60.4 and 58.6 pg/mL, respectively, compared with 86.4 pg/mL for vitamin D ≤ 20 ng/mL). This suggests that clinically important elevations in PTH tend to be observed in vitamin D deficiency rather than vitamin D insufficiency. Consistent with our findings, large increases in the upper reference limit for PTH were found previously for vitamin D levels ≤ 12 ng/mL in a cohort of women aged 17–84 years, where 25(OH)D levels were analyzed by isotype dilution liquid chromatography–tandem mass spectrometry, and PTH levels were evaluated using the Elecsys^®^ PTH assay [22]. The results do not seem to be population specific, since race, season, and latitude do not add to the ability to predict PTH over that of 25(OH)D alone.

The dominant covariate associated with PTH was vitamin D level. A relationship between race and PTH was also observed, but it was complex. Overall, the addition of race to a statistical model predicting PTH using vitamin D levels did not improve the model, indicating that separate reference ranges by race may not be required. However, previous studies suggest that African American individuals manifest several differences in calcium-related pathways compared with white individuals [23]. In the present analyses, black/African Americans had higher levels of PTH and lower levels of vitamin D compared with Caucasian individuals, with the greatest difference in PTH observed among vitamin D-deficient individuals. In addition, a statistically significant interaction was found between race and vitamin D in predicting PTH levels, indicating that effects of vitamin D on PTH differ between races. These findings are consistent with recent data showing similar findings, and suggest that vitamin D bioavailability or metabolism may be different in African Americans compared with Caucasians [23, 24]. Season and latitude were generally not predictive of PTH levels, but other differences between collection sites were identified [25, 26].

Limitations of the study include a gender imbalance (protocol-specified: 50 ± 5% not achieved), and predominance of individuals aged < 60 years; so results may not be generalizable to older individuals in whom elevated PTH concentrations have been observed [27]. Ethnic differences in PTH levels have previously been reported for Asian individuals; our results may, therefore, not be applicable to regions with larger Asian populations, as most individuals enrolled were Caucasian (68.2%) or black/African American (28.6%) [28]. However, the study population is reflective of USA sample collection sites and consistent with an earlier FDA request to include 30% black/African American individuals for determination of expected values for 25(OH)D. Previous studies have shown good analytical performance for both the Elecsys^®^ Vitamin D total II and PTH assays; recently, Batista et al. [29] found good agreement between the Elecsys^®^ Vitamin D total II assay and LC–MS/MS (90.8%), with mean imprecision and inaccuracy below 10% and 5%, respectively, as recommended by the Vitamin D External Quality Assessment Scheme (DEQAS) [30]. Furthermore, the Elecsys^®^ Vitamin D total II assay has received CDC certification [31], and demonstrates excellent comparability with CDC verification samples that have concentrations assigned by the CDC vitamin D reference laboratory using ID-LC–MS/MS (Deming regression: *y* = 0.954*x* − 0.707; *r* = 0.982) [32]. The assay also shows excellent comparability with DEQAS samples that have assigned target values from the US National Institute for Standards and Technology [NIST; sample numbers 476–490; mean recovery 100% (84–110%); mean relative bias − 0.4%] [32]. For the Elecsys^®^ PTH assay, Hermsen et al. [33] found coefficients of variation of ≤ 6.6% and ≤ 15.6% for within-run and between-day precision, respectively.

Key strengths of the study include conduct of analyses in accordance with CLSI guidelines and classification of vitamin D-replete individuals based on a 25(OH)D serum concentration of ≥ 30 ng/mL, as opposed to > 20 ng/mL in earlier studies; the higher threshold may be more relevant to patient populations than to the general population [9, 17].

This study provides new reference ranges for the Elecsys^®^ PTH assay, determined in serum from a large population of apparently healthy individuals, stratified according to three categories of 25(OH)D concentration. The availability of robust reference ranges accounting for 25(OH)D status is likely to facilitate clinical decision-making.
